# Genetic Network between Leaf Senescence and Plant Immunity: Crucial Regulatory Nodes and New Insights

**DOI:** 10.3390/plants9040495

**Published:** 2020-04-13

**Authors:** Yi Zhang, Hou-Ling Wang, Zhonghai Li, Hongwei Guo

**Affiliations:** 1 Beijing Advanced Innovation Center for Tree Breeding by Molecular Design, Beijing Forestry University, Beijing 100083, China; yizhang@bjfu.edu.cn (Y.Z.); whling@bjfu.edu.cn (H.-L.W.); 2 Institute of Plant and Food Science, Department of Biology, Southern University of Science and Technology (SUSTech), Shenzhen 518055, China

**Keywords:** leaf senescence, plant immunity, hormone, Sen-TF, cross-regulation

## Abstract

Leaf senescence is an essential physiological process that is accompanied by the remobilization of nutrients from senescent leaves to young leaves or other developing organs. Although leaf senescence is a genetically programmed process, it can be induced by a wide variety of biotic and abiotic factors. Accumulating studies demonstrate that senescence-associated transcription factors (Sen-TFs) play key regulatory roles in controlling the initiation and progression of leaf senescence process. Interestingly, recent functional studies also reveal that a number of Sen-TFs function as positive or negative regulators of plant immunity. Moreover, the plant hormone salicylic acid (SA) and reactive oxygen species (ROS) have been demonstrated to be key signaling molecules in regulating leaf senescence and plant immunity, suggesting that these two processes share similar or common regulatory networks. However, the interactions between leaf senescence and plant immunity did not attract sufficient attention to plant scientists. Here, we review the regulatory roles of SA and ROS in biotic and abiotic stresses, as well as the cross-talks between SA/ROS and other hormones in leaf senescence and plant immunity, summarize the transcriptional controls of Sen-TFs on SA and ROS signal pathways, and analyze the cross-regulation between senescence and immunity through a broad literature survey. In-depth understandings of the cross-regulatory mechanisms between leaf senescence and plant immunity will facilitate the cultivation of high-yield and disease-resistant crops through a molecular breeding strategy.

## 1. Introduction

The leaf is the organ that uses the photosynthetic system to convert light into sugars, thus providing energy for plant growth and development. As leaf age increases, the photosynthesis ability gradually decreases and then enters its final stage: leaf senescence [1]. In *Arabidopsis*, leaf senescence occupies nearly half of the leaf life history from the onset of senescence to completely dead [2]. Although leaf senescence is basically controlled by developmental age, it can be induced by a variety of internal signals, including phytohormones and reproduction, and external factors, such as darkness, UV-B or ozone, nutrient limitation, heat or cold, drought, high salinity, and pathogen attacks [2]. Leaf senescence is critical for plants’ fitness and survival because plants reallocate nutrients from senescent leaves to young leaves and other organs during age- or stress-induced senescence [3,4].

Various molecular, physiological, and biochemical events in cells are genetically regulated in an orderly manner in the entire developmental stage of leaves. Cell cycle, cell wall biogenesis, macromolecule biosynthesis, and photosynthesis are active in the stage before the initiation of senescence. Nevertheless, cell wall disassembly, anthocyanin biosynthesis, and amino acid transport mainly occur during leaf senescence [2]. The metabolic and gene expression profile of cell changes orderly when the leaf senescence is initiated. In addition, the contents of phytohormones, such as ethylene (ET), jasmonates (JA), salicylic acid (SA), and abscisic acid (ABA) increase as leaf ages, whereas gibberellic acid (GA) and cytokinin (CK) decrease, suggesting that they play differential roles in regulating leaf senescence. Reactive oxygen species (ROS) accumulate as leaf ages [5] and function as positive players in leaf senescence [6]. Eventually, leaf senescence displays a programmed cell death (PCD)-like event, which is also termed as developmentally controlled PCD (dPCD) [7].

Plants respond to pathogen attacks using an innate immune system that is broadly divided into pathogen-associated molecular patterns (PAMP)-triggered immunity (PTI) and effector-triggered immunity (ETI). PTI is initiated by which plants respond to PAMP through cell surface-localized pattern recognition receptors (PRRs) [8]. PTI causes rapid defense responses, such as cell wall enhancement, ion flux, ROS production, mitogen-activated protein kinase (MAPK) cascades, hormone networks, and the expression of defense-related genes [9]. However, successful pathogens can secrete effectors to interfere with PTI signaling pathways. In turn, the hosts use intracellular receptors to recognize effectors, resulting in ETI [8]. ETI usually causes enhanced defense response, such as hypersensitive response (HR), which is a form of PCD.

Plant pathogens are generally divided into biotrophs and necrotrophs according to their lifestyles [10]. Biotrophic pathogens feed nutrients from living host tissues, whereas necrotrophic pathogens take nutrients from dead cells destroyed by their secreted phytotoxins and cell-wall-degrading enzymes. In addition, some pathogens are called hemibiotrophs, because they apply two lifestyles during infection. In plant-pathogen interactions, pathogen-triggered PCD (pPCD) is an effective strategy to limit pathogen spread, especially for biotrophic pathogens. However, pPCD would cause nutrient leakage, which is a benefit for necrotrophic pathogens growth. For pathogens, biotrophic pathogens repress the cell death process, whereas the necrotrophic pathogens promote this case. Thus, plants manipulate their cell death process to cope with pathogens with different lifestyles [11].

PCD is also a typical characteristic of leaf senescence. Although there are many differences between dPCD and pPCD, they are controlled by partial common components [12]. Intriguingly, dPCD-accelerated plants generally show enhanced resistance against biotrophic and hemibiotrophic pathogens, but impaired resistance to necrotrophic pathogens. Nevertheless, dPCD-delayed plants are the opposite (Summarized in Section 3). dPCD-accelerated plants are usually accompanied with higher accumulation of SA and/or ROS. SA and ROS are closely linked to PCD and they are required for plant resistance against biotrophic and hemibiotrophic pathogens. Moreover, they are required for the establishment of both dPCD and pPCD [13]. Thus, SA, ROS, as well as PCD, seem to function as key nodes connecting leaf senescence and plant immunity. In this paper, we review the regulatory roles of SA and ROS on leaf senescence and plant immunity, summarize the transcriptional controls of Sen-TFs on SA and ROS signal pathways, and outline the cross-regulation between leaf senescence and plant immunity by manual curation.

## 2. The Roles of SA and ROS in Leaf Senescence and Plant Immunity

SA is a well-known hormone for plant disease resistance and is required for systemic acquired resistance (SAR), and plays an important role in the amplification of PTI and ETI [14]. SA-induced and age-dependent gene expression changes exhibit a high degree of overlap in genome-wide transcriptome analysis [2], indicative of the dual roles of SA in leaf senescence and plant immunity. Isochorismate synthase (ICS) mainly synthesizes SA. *Arabidopsis* genome contains two ICS genes, *ICS1* (also known as *SID2*) and *ICS2*. Age- and pathogen-induced SA is synthesized through the ICS1 [5,15]. *ICS1* is rapidly induced by pathogen attack and it is essential for the establishment of SAR [15,16]. Different from ICS1, ICS2 contributes to the synthesis of basal SA in plants [17]. The mutation in *ICS1* leads to more susceptible to *Pseudomonas syringae* pv. *tomato* (*Pst*) pathogens [15]. However, the local resistance against *Botrytis cinerea* is involved in SA in an ICS1-independent manner [18]. Moreover, the *sid2* mutants display delayed leaf senescence phenotypes as compared to Col-0 plants [5]. SA is further perceived by NPR1 (Non-expressor of PR gene 1), which is regarded as a SA receptor demonstrated by recent studies and is responsible for the SA signaling pathway [19,20]. Mutation in NPR1 impairs its function in promoting SA-induced defense gene expression and thereby causes decreased resistance to pathogens [20]. However, the resistance of *npr1* mutants to necrotroph *Alternaria brassicicola* shows a similar level when compared with WT plants [21]. The function of NPR1 in regulating leaf senescence is obvious, as the mutation of NPR1 delays the reaching of chlorotic stage [22]. *SAG12*, which is a marker gene of leaf senescence, is considerably reduced in *npr1* mutants compared to Col-0 plants. In addition, EDS1 (ENHANCED DISEASE SUSCEPTIBILITY1) and PAD4 (PHYTOALEXIN DEFICIENT4) form a heterodimer to regulate plant immunity and are indispensable for the accumulation of SA induced by virulent pathogens [23]. EDS1 and PAD4 positively regulate disease resistance against *Pst* pathogens [23], but virus-induced gene silencing of *EDS1* enhances the tobacco resistance to *B. cinerea* [24]. Similar to *npr1*, the *pad4* mutant exhibits delayed senescence phenotypes when compared with Col-0 plants [22], and PAD4 positively modulates green peach aphid feeding-induced leaf senescence [25]. The transcriptional level of *ICS1* increases during leaf senescence. Consistently, SA content accumulates as leaf aging and has been used as an important inducer of leaf senescence [5]. Thus, the SA signaling pathway plays critical roles in both plant immunity and leaf senescence.

ROS play an integral role in the regulation of numerous biological processes, including leaf senescence and responses to pathogen attacks [13]. ROS are defined as oxygen-containing molecules with higher chemical reactivity than O_2_. The major forms of ROS in plants include superoxide anion, singlet oxygen, hydroxyl radical, and hydrogen peroxide (H_2_O_2_). Among them, H_2_O_2_ is the most stable and generally acts as a signal molecule to trigger downstream responses. ROS can be produced in various subcellular compartments, such as plasma membrane, cell wall, mitochondria, chloroplasts, and so on [26]. In general, intracellular ROS are produced in chloroplasts, mitochondria, and peroxisomes, and the apoplastic ROS are produced by plasma membrane-localized NADPH oxidases (RBOHs, respiratory burst oxidase homologs) and cell wall peroxidases, under both normal and stress conditions [26]. ROS act as double-edged swords in cellular processes. Low level of ROS functions as signaling molecules in response to biotic and abiotic stresses, while a high level of ROS causes PCD or even necrosis [27]. In plant-pathogen interaction, an increased production of ROS occurs during PTI and ETI after pathogen recognition and generally contributes to disease resistance. Furthermore, early accumulated ROS could directly strengthen the cell wall and act as signal molecules to activate defense responses [13]. ROS act in combination with SA to regulate the SAR establishment. Changes in the redox status cause the movement of NPR1 to the nucleus to induce defense gene expression [15]. A number of key senescence-associated genes (SAGs), such as *ORE1* [28], *WRKY53* [29], and *WRKY75* [5], are transcriptionally up-regulated in response to H_2_O_2_ stimulus. ROS also accumulate during senescence and function as positive players of leaf senescence [5,6].

SA can induce the accumulation of H_2_O_2_, and vice versa. SA directly inactivates H_2_O_2_ scavengers, such as catalases (CATs) and ascorbates peroxidases (APXs), resulting in the accumulation of H_2_O_2_ [30]. Conversely, the exogenous application of H_2_O_2_ could induce SA biosynthesis in tobacco [31]. Furthermore, SA and ROS act synergistically to trigger both dPCD and pPCD [12]. PCD plays an essential role in immunity and development. However, dPCD exhibits some distinct characteristics that differ from pPCD. dPCD is an active and long-term process, while pPCD is a passive and rapid process. HR, which is the best-characterized pPCD, is an efficient strategy against pathogen attack. Pathogenesis-related (PR) genes are associated with HR, and *HIN1* (*hairpin-induced gene 1*) is an HR marker gene [32]. Notably, these genes are also induced in an age-dependent manner [33], indicating that some common pathways or cross-talks exist between dPCD and pPCD at the molecular level.

## 3. The Regulators of SA and ROS

### 3.1. Transcription Factors

**Figure 1:**
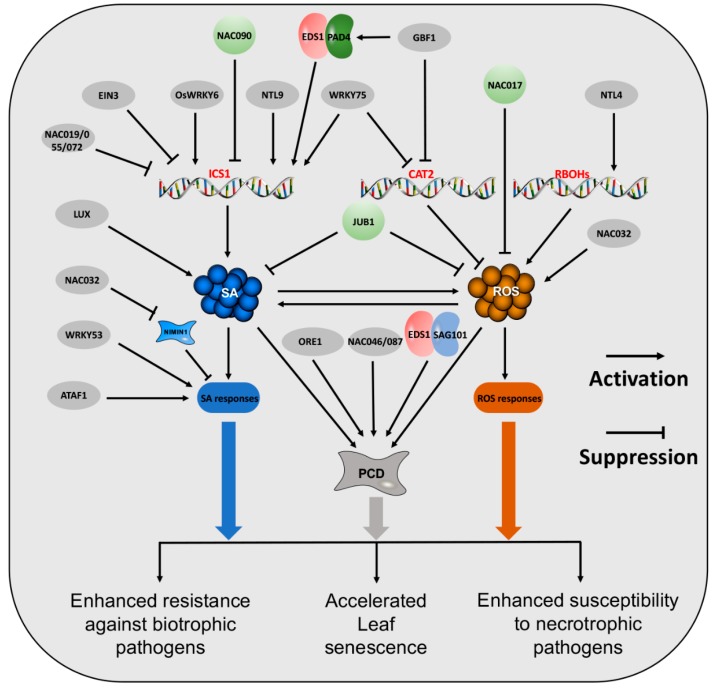
Cross-Regulation Network between Leaf Senescence and Plant Immunity Mediated by Salicylic Acid (SA) and Reactive Oxygen Species (ROS). SA, ROS, and programmed cell death (PCD) play a positive role in the regulation of leaf senescence and resistance against biotrophic pathogens but a negative function for plant resistance to necrotrophic pathogens. TFs that positively regulate SA, ROS and/or PCD, including WRKY75, NTL9, GBF1, and ORE1, also positively regulate leaf senescence and plant immunity. Negative regulators, such as JUB1, represses SA and ROS as well as leaf senescence and plant immunity. EIN3 and NAC019/055/072 directly bind to the promoter of *ICS1* and suppress its expression, resulting in a decrease of SA production. Abbreviations: LUX, LUX ARRHYTHMO; NAC, NAM/ATAF/CUC; EIN, ETHYLENE INSENSITIVE; EDS, ENHANCED DISEASE SUSCEPTIBILITY; PAD, PHYTOALEXIN DEFICIENT; WRKY; GBF1, G-BOX BINDING FACTOR 1; NTL, NAC with transmembrane motif 1-like; JUB1, JUNGBRUNNEN1; SA, salicylic acid; ROS, reactive oxygen species; NIMIN, NIM1 INTERACTING; ORE, ORESARA; SAG, Senescence-associated Gene; ATAF, ACTIVATING FACTOR; PCD, programmed cell death; CAT2, CATALASE 2; ICS, isochorismate synthase; RBOH, respiratory burst oxidase homolog.

The *Arabidopsis* developmental transcriptome based on RNA-seq profiling reveals that a large number of genes are upregulated during senescence [34], which are SAGs. Molecular genetic studies demonstrate that numerous SAGs function as key regulators of leaf senescence [35,36]. Intriguingly, many SAGs have also been reported to be implicated in plant immunity. Among them, NAC, WRKY, TCP, MYB, and bZIP family members are associated with leaf senescence and plant resistance, suggesting a pivotal significance of Sen-TFs in the regulation of these two processes [36,37]. As SA and ROS play important roles in the regulation of leaf senescence and plant disease resistance, and ICS1, RBOHs and CAT2 control age- and pathogen-induced SA and ROS biosynthesis and/or metabolism, respectively, they are subjected to complex regulation by Sen-TFs at the transcriptional level (Figure 1).

#### 3.1.1. NAC TFs

The plant-specific NAC TF family has more than 100 members in *Arabidopsis* [38], and many of them are involved in the regulation of leaf senescence and plant immunity (Table 1). Some positive regulators of leaf senescence, such as ORE1 [28], NTL9 [39], NAC032 [40]], and ATAF1 [41], also play important roles in plant immunity. The mutations of these genes delay leaf senescence, while overexpressing plants display precocious senescence phenotypes. Moreover, these mutants support a higher level of hemibiotrophic pathogens, such as *Pst* DC3000. The overexpression of these genes confers plant resistance against *Pst* DC3000, but susceptibility to the necrotrophic pathogens, such as *B. cinerea* [42,43,44,45]. ORE1 has been shown to mediate PCD via directly targeting and activating a senescence-enhanced gene *BIFUNCTIONAL NUCLEASE 1* (*BFN1*), which is associated with PCD [46]. Similarly, NAC087/046 control dPCD by regulating *BFN1* in *Arabidopsis* lateral root cap [47]. Furthermore, BnaNAC87, the homolog of NAC087 in *Brassica napus*, acts as a positive regulator of ROS metabolism and cell death [48] and it binds the promoter of *HIN1* [32,48]. However, the function of NAC087/046 in plant immunity remains unknown. The *P. syringae* type III effector HopD1 could target NTL9 to suppress ETI and contributes to its virulence [43], indicating that NTL9 is an important component in *Arabidopsis* immune system against *Pst* pathogens. Indeed, NTL9 directly binds the promoter of *ICS1* in yeast one-hybrid assays and it is required for the induction of *ICS1* by flg22 treatment [49]. ANAC032 activates SA signaling by repressing NIMIN1, a key negative regulator of SA-dependent defense, and promotes H_2_O_2_ accumulation under stress [40,45]. Additionally, the overexpression of *Gossypium hirsutum ATAF1* in cotton also activates SA signaling [44], although the mechanism is unclear.

JUNGBRUNNEN1 (JUB1, NAC042) is an H_2_O_2_-induced NAC TF and functions as a negative regulator of longevity in *Arabidopsis* [6]. The overexpression of *JUB1* causes the accumulation of DELLA proteins and antagonizes SA pathway, resulting in impaired resistance to *Pst* DC3000, but increases resistance against necrotrophic fungus *A. brassicicola* [55]. Additionally, higher H_2_O_2_ accumulation is observed in *jub1* mutants compared to Col-0 plants, whereas overexpressing *JUB1* could decrease the cellular H_2_O_2_ level, suggesting that JUB1 is involved in the regulation of biosynthesis and/or metabolism of H_2_O_2_ [6]. Recently, NAC017, NAC082, and NAC090 have been reported to govern the positive-to-negative regulatory shift in leaf senescence, which is referred to as a “NAC troika”. A single mutant of *nac017*, *nac082*, or *nac090* accelerates cell death and leaf senescence, while overexpressing lines show the opposite effect, indicative of their negative regulatory roles in leaf senescence [57]. NAC090 exhibits a predominant role in repressing SA accumulation and responses by directly binding the promoters of target genes *ICS1* and *EDS5*. Different from NAC090, ANAC017 predominantly suppresses ROS levels.

NAC019, NAC055, and NAC072 (RD26) positively regulate age-dependent leaf senescence [58]. However, their roles in plant immunity are complicated. The *anac019 anac055 anac072* triple mutant shows increased resistance to *P. syringae* pv. *maculicola* (*Psm*) ES4326 [60]. In contrast, *anac019 anac055* double mutant exhibits enhanced resistance to necrotrophic fungus *B. cinerea*, while the overexpression of *ANAC019* or *ANAC055* has the opposite effects [61]. ANAC019, ANAC055, and ANAC072 function redundantly in the suppression of SA accumulation by repressing the expression of *ICS1* [49], suggesting their promotive role of leaf senescence in a SA-independent manner. Indeed, ANAC019 and ANAC055 function as transcription activators to regulate the expression of JA-related defense genes in a COI1- and MYC2-dependent manner [61].

In addition to the above-mentioned transcription factors, several NAC TFs, such as NAC059 (ORS1) [62] and NTL4 [63], have only been implicated in the positive regulation of leaf senescence, which cannot rule out the possibility that they are also involved in plant immunity. Microarray-based expression profiling by using estradiol-inducible *ORS1* overexpression lines reveals that several potential targets of ORS1, such as *WRKY40* [74], *WRKY75* [5], *FMO1* [87,88], and *ALD1* [89], play critical roles in plant immunity. ChIP assays show that NTL4 induces the accumulation of ROS by directly targeting the promoters of ROS biosynthesis-related genes RBOHs to control PCD during heat- and drought-induced leaf senescence [63,64], which implies that NTL4 is involved in drought/heat stress-triggered disease resistance.

#### 3.1.2. WRKY-TFs

WRKY TFs are one of the largest families of plant-specific TFs, with more than 70 members in *Arabidopsis* [90]. Although WRKY family TFs are well known for their functions in plant immunity, they are also implicated in the regulation of leaf senescence (Table 1). WRKY53, WRKY75, WRKY22, *Oryza sativa* WRKY6 (OsWRKY6), and *Chimonanthus praecox* WRKY71 (CpWRKY71) are positive regulators of leaf senescence [5,29,69,70,91] and/or disease resistance against hemibiotrophic pathogens [5,65,69]. Of them, WRKY53, WRKY75, and OsWRKY6 are involved in the regulation of SA and/or ROS signaling (Figure 1). WRKY53 is partially involved in the SA-signaling pathway [92], and it interacts with the JA-inducible protein EPITHIOSPECIFYING SENESCENCE REGULATOR (ESR/ESP) to antagonistically regulate SA-JA signaling during leaf senescence [93]. WRKY75 promotes SA production by directly binding to the W-box (TTGACT) sequence in the promoter of *ICS1* to activate its transcription. Moreover, WRKY75 suppresses the transcription of *CAT2* to repress H_2_O_2_ scavenging, which results in an accumulation of ROS level. OsWRKY6 directly binds and positively regulates *OsICS1* to enhance SA accumulation, leading to constitutive activation of several PR genes [69].

As a negative regulator of leaf senescence, WRKY18 directly binds the W-boxes of *WRKY53* promoter and suppresses its expression [72]. WRKY18 acts with its closely related homologs WRKY40 and WRKY60 to redundantly regulate disease resistance. The double mutants *wrky18 wrky40* and *wrky18 wrky60*, as well as the triple mutant *wrky18 wrky40 wrky60* display enhanced resistance to *P. syringae*, but are more susceptible to *B. cinerea* than Col-0 plants. Moreover, the double mutant *wrky18 wrky40* is more resistant to the biotrophic powdery mildew fungus *Golovinomyces orontii* [73,74]. The triple mutant *wrky18 wrky40 wrky60* exhibits a higher level of SA-responsive gene *PR1*, implying that they negatively regulate SA signaling.

WRKY70 and WRKY54 act redundantly to negatively regulate leaf senescence in *Arabidopsis* [66], functioning as negative regulators of SA biosynthesis, but positive regulators of SA-mediated gene expression [94]. WRKY70 acts as a common component in both SA- and JA-mediated signal pathways, which is activated by SA and repressed by JA [95]. The elevated expression of *WRKY70* results in impaired resistance to necrotroph *A. brassicicola*, whereas enhances the resistance against biotroph *Erysiphe cichoracearum* [67], suggesting the dual roles of WRKY54 and WRKY70 in repressing SA biosynthesis and transducing the SA signal.

#### 3.1.3. Other TFs

In addition to NAC and WRKY TF families, other TF family members have also been reported to regulate leaf senescence and plant immunity (Table 1). GBF1, a bZIP protein, controls leaf senescence by directly regulating PAD4 [76] and binding the promoter of *CAT2* to suppress its transcription, thus resulting in the decreased H_2_O_2_-scavenging activity [75]. Accordingly, the *gbf1* mutant shows a decreased H_2_O_2_ level and delayed senescence phenotypes [75]. The *GBF1* overexpressing plants are more resistant, whereas *gbf1* is susceptible, to *Pst* pathogens when compared to Col-0 [76]. The circadian clock pathway is known to regulate leaf senescence and plant innate immunity [77,96]. One of the core clock components LUX (LUX ARRHYTHMO, also called PHYTOCLOCK1), an MYB family TF, is essential for circadian rhythmicity that functions as a negative regulator of leaf senescence. The leaf of *lux-2* shows earlier yellowing as compared with Col-0 [77], and *lux* mutants display impaired disease resistance against *P. syringae* and SA- and JA-mediated defense signaling [78]. TCP TFs constitute a small family of plant-specific TFs that play important roles in plant development [97]. The *tcp19* or *tcp20* single mutant shows slightly early senescence phenotype in the dark, whereas *tcp19 tcp20* double mutant displays significantly accelerated senescence, suggesting that TCP19 and TCP20 redundantly regulate leaf senescence [79]. Although the resistance phenotype of *tcp19 tcp20* has yet to be studied, the TCP19 was found to bind the promoters of *ICS1*, *PBS3* (*avrPphB SUSCEPTIBLE 3*), *PAD4*, and *EDS1* (ENHANCED DISEASE SUSCEPTIBILITY 1), indicative of their possible involvement in plant immunity [49].

### 3.2. Non-TFs

In addition to TFs, other types of genes are also involved in the regulation of leaf senescence and disease resistance (Table 1). *SAG101*, a gene encoding an acyl hydrolase, plays a positive role in leaf senescence [80]. SAG101 has been demonstrated to function as an important component in plant immunity by forming a heterodimer with EDS1, thus mediating TNL-mediated PCD [23]. *Arabidopsis HLS1* (*HOOKLESS1*) encodes a putative histone acetyltransferase and negatively regulates dark-, pathogen-, and ABA-induced leaf senescence [81]. A higher ROS level is observed in ABA-induced senescing leaves in the *hls1* mutant. The loss of HLS1 function leads to an increased susceptibility to *B. cinerea* with larger disease lesions, enhanced necrosis, and chlorosis when compared to controls [81]. However, *hls1* mutants also display enhanced disease symptoms after inoculation with *Pst* DC3000 (*AvrRpm1*), but they show a level of bacterial growth under *Pst* DC3000 inoculation, suggesting that HLS1 manipulates defense responses associated with ETI [81]. JMJ16 is a JmjC domain-containing protein and it is a specific H3K4 demethylase in *Arabidopsis*. The expression of two positive regulators of leaf senescence, *WRKY53* and *SAG201*, is repressed by JMJ16 in an age-dependent manner through reducing H3K4me3 levels at these loci [82]. The *jmj16* mutants display an early senescence phenotype, suggesting that JMJ16 is an important epigenetic regulator of leaf senescence via demethylating H3K4 at *SAG*s in an age-dependent manner. RNA_seq and ChIP_seq reveal that lots of potential target genes of JMJ16, such as *WRKY33*, *CBP60G*, *EDS5*, and *PR5*, are associated with plant immunity. However, the disease resistance phenotype of *jmj16* is not reported so far.

Two *Arabidopsis* MAPKs, MPK3 and MPK6, play crucial roles in response to biotic and abiotic stresses. MPK3/6 are involved in PTI and ETI [84], and *mpk6* mutants show delayed senescence phenotypes [83]. MAPK phosphatase 2 (MKP2) could functionally interact with MPK3/6 and dephosphorylate them, and regulate oxidative stress as well as pathogen defense responses [85]. Contrary to the *mpk6* mutants, the MKP2-suppressed line (*MPK2_RNAi*) displays early senescence phenotypes [35,83]. Another protein phosphatase, phosphatase 2A subunit PP2A-B′γ, was recently reported to negatively regulate leaf senescence and resistance against necrotroph *B. cinerea*, suggesting that MKP2 and PP2A regulate disease resistance against necrotrophs in different mechanisms [98].

*Arabidopsis* FtSH4, a mitochondrial AAA-protease, mediates autophagy and senescence [86], and the *ftsh4* mutant shows a significantly increased SA level and accelerated leaf senescence and cell death. Although the function of FtSH4 in disease resistance has not been studied, several SA biosynthesis and signaling genes, such as *SID2*, *NDR1*, and *NPR1*, are upregulated in the *ftsh4* mutant. Furthermore, the loss of SID2, NDR1, or NPR1 functions in the *ftsh4* mutant restores the early senescence phenotypes. In addition, several WRKY genes, including *WRKY40*, *WRKY46*, *WRKY51*, *WRKY60*, *WRKY63*, and *WRKY75*, display elevated expression levels in *ftsh4* mutant. Together, the above data imply that FtSH4 plays a negative role in plant immunity.

### 3.3. The Differences between Age- and Pathogen-Induced SA and ROS

Age- and pathogen-induced changes in SA and ROS can be regulated by a large number of Sen-TFs, as mentioned above. Although Sen-TFs are involved in these two processes, there are still significant differences between them. The spatial and temporal expression of Sen-TFs is strictly regulated, and their transcript levels are gradually upregulated during leaf senescence. Consistently, the contents of SA and ROS gradually increase as the leaf ages. By contrast, pathogen-evoked acute induction of Sen-TFs gene expression as well as the contents of SA/ROS would usually return to basal levels after a certain period of time.

The physiological state of plants, such as age, has complex and delicate regulation in gene expression. For example, the “NAC troika” precisely governs the time-dependent regulatory shift for NAC-TFs in *Arabidopsis* during leaf senescence. In addition to age, the functional differences of the common regulatory components of SA and ROS are likely dependent on the tissue specificity of gene expression. For instance, NTL9 functions in guard cells for flg22-triggered induction of SA synthesis- related genes and is required for stomatal immunity [49]. However, NTL9 might regulate leaf senescence by regulating the expression of a subset of *SAG*s in other cell types, such as mesophyll cells. Multi-omics analysis of specific cell types (single-cell sequencing) would contribute to the understanding of the functional differences of Sen-TFs on leaf senescence and plant immunity.

## 4. Cross-Talks between SA/ROS and Other Hormones during Leaf Senescence and Plant Immunity

**Figure 2:**
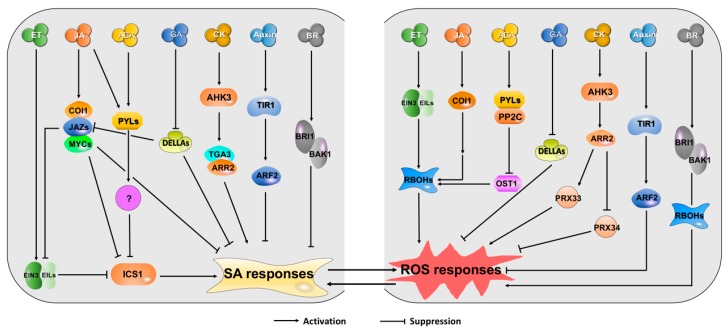
SA and ROS Are the Hubs that Integrate Regulatory Signals from Plant Hormone Pathways to Control Leaf Senescence and Plant Immunity. Cross-talks between SA/ROS and other hormones are mainly modulated by key components in their signaling pathways. The SA-dependent responses are suppressed by JA, ET, and ABA via repressing SA-related genes such as biosynthesis gene *ICS1*. Auxin and BRs also antagonize SA signaling pathway. Additionally, GA and CK mainly synergistically interact with SA. Meanwhile, besides Auxin, ET, JA, ABA, GA, CK, and BR can contribute positively to ROS responses. SA can trigger the accumulation of ROS, and vice versa. Abbreviations: ET, ethylene; JA, jasmonic acid; ABA, abscisic acid; GA, gibberellin; CK, Cytokinin; BR, Brassinosteroid.

Other phytohormones generally regulate senescence and immune responses by antagonistically or synergistically interacting with the SA/ROS signaling pathway because SA and ROS act as central players in leaf senescence and plant immunity [99]. Cross-talks between SA/ROS and other hormones are discussed here, and the key components of plant hormone pathways involved in leaf senescence and/or plant immunity are summarized in Table 2 and shown in Figure 2.

ET synergistically interacts with JA in plants in response to stresses and generally antagonizes SA signaling [128]. However, the molecular details of the antagonism of JA/ET signaling on the SA pathway are still poorly understood. JA signal is perceived through JA receptor complex that constituted the F-box protein CORONATINE INSENSITIVE 1 (COI1) and JASMONATE ZIM-domain (JAZ) proteins, and activates various downstream TFs, including the IIIe bHLH TFs MYC2, MYC3, and MYC4. *Pst* pathogen secretes a mimic of JA-Ile virulence factor coronatine (COR) to suppress SA-dependent defenses by activating MYC2 and NAC019/055/072, which repress SA synthesis and/or promote SA metabolism [60]. Consistently, an elevated level of SA is found in the *coi1* mutant [107]. JA induces stomatal closure mediated by ROS-dependent signaling pathway. JA increases the ROS level via the NAD(P)H oxidase pathway, but JA-induced ROS accumulation is not observed in *coi1* or *jar1-1* mutants [129]. ET is perceived by its receptors and then leads to the activation of ETHYLENE-INSENSITIVE 2 (EIN2) [130] and the core TFs EIN3/EIL1 (EIN3-LIKE 1) [131]. EIN3/EIL1 thus activate a large number of downstream genes. For example, EIN3/EIL1 directly target the promoter of *SID2* and negatively regulate the accumulation of SA. The *ein3 eil1* double mutant thus constitutively accumulates SA, and ROS production is diminished in ethylene-insensitive mutants under Flg22 treatment [132]. In rice, OsEIL1 directly binds the promoters of *OsrbohA/OsrbohB* to activate their transcription [133]. ET synergistically acts with JA to activate the expression of *ERF1* in response to necrotrophic pathogens [99]. JA promotes ABA signaling by inducing the expressions of ABA receptors, such as PYL4 (PYRABACTIN RESISTANCE/PYR1-LIKE/REGULATORY COMPONENT OF ABA RECEPTOR (PYR/PYL/RCAR)). Conversely, ABA promotes JA signaling by regulating the expressions of *MYC* TFs in response to the herbivorous insect [134,135]. As the key components of GA signaling, DELLA proteins interact with JAZ1 to activate MYC-dependent gene expressions in response to stresses [136]. Auxin enhances JA-induced expressions of defense-related genes and JA-Auxin module is involved in resistance to necrotrophic pathogens [137]. However, JA inhibits apical growth of roots and auxin has the opposite effect, suggesting they antagonistically regulate root growth. A possible model is that JA represses the expressions of auxin-responsive TFs, *PLETHORAs* (*PLTs*), which are responsible for stem cell niche maintenance [138]. JA antagonistically interacts with CKs to regulate xylem development. CKs negatively regulate xylem differentiation, while JA treatment promotes the formation of extra xylem [139].

Recent studies have greatly expanded our understandings of the cross-talks between ABA and other hormones. ABA antagonizes plant immune responses by repressing SA responses. The *ICS1* transcriptional level is down-regulated in ABA receptor mutant *pyl*. In rice, ABA significantly suppresses the expression levels of *OsWRKY45* and *OsNPR1* [140]. Meanwhile, increasing evidence indicates that ABA also regulates ROS production through RBOHF. In the presence of ABA, PYLs inhibit PP2Cs, resulting in activation of one of SNF1-related protein kinases (SnRK2s), OPEN STOMATA 1 (OST1), to phosphorylate the N terminus of RBOHF, thus increasing the ROS level [141]. MYC2 is a critical and positive node in the crosstalk between JA and ABA signaling pathways. ABA interacts with JA signaling via PYL6-MYC2 to regulate cotyledon expansion [142] and the tolerance to drought [143]. Moreover, the Plant U-box (PUB) E3 ligase PUB10 directly interacts with MYC2 to destabilize it, negatively regulating both JA and ABA responses [144]. The antagonistic interactions between ABA and ET have been reported in seed dormancy and germination [145]. One of the mechanisms is that ABA inhibits the transcript level and activity of ACO1 [146]. The SnRK2-APC/C^TE^ module is a regulatory hub that is involved in the cross–talks between GA and ABA [147]. Rice *Tiller Enhancer* (*TE*), encoding an activator of the APC/C^TE^ E3 ubiquitin ligase complex, interacts with and degrades ABA receptor OsPYLs. SnRK2 phosphorylates TE to interrupt the interaction between TE and OsPYLs. GA reduces SnRK2s levels and inhibits APC/C^TE^-mediated degradation of OsPYLs. ABI4 positively regulates ARR5 (Arabidopsis RESPONSE REGULATOR 5) expression, a negative regulator of CK signaling, but it suppresses the transcript level of *PIN-FORMED 1* (*PIN1*), indicating that ABI4 mediates the cross-talks among ABA, CK, and auxin [148]. SnRK2.3 interacts with and phosphorylates HAT1, an important transcriptional regulator in BR signaling, to repress its protein stability and binding activity, which increases expressions of ABA-responsive genes and then enhances tolerance to drought, indicating that a relationship between ABA and BR signaling pathways exists [149]. The cross-talks between ABA and other hormones in plant stress responses are discussed in details in several recently published papers [150,151].

GAs, CKs, auxins, and BRs also play important roles in plant development and immune system. GAs regulate these two processes by mediating the degradation of DELLA proteins [152]. DELLAs sequester the JA signaling repressor JAZ family proteins, leading to the activation of MYC2 and JA signaling. Meanwhile, DELLAs also directly antagonize the SA-mediated defense [120]. The accumulation of DELLAs that is induced by stresses causes the enhanced activity of ROS scavenging enzyme and suppresses ROS levels [153]. CKs are reported as modulators of plant immunity by interacting with SA. ARR2, a key TF of CK signaling pathway, interacts with TGA3 and positively enhances the expression of *PR1* gene, suggesting that CKs can also function in the SA signaling pathway [154]. CKs receptor HISTIDINE KINASE3 (AHK3) and ARR2 promote PAMP-triggered stomatal closure and ROS accumulation. ARR2 directly activates the expressions of peroxidases PRX33 and PRX34, but not RBOHs, which are required for SA- and PAMP-triggered ROS production [122]. Auxins regulate plant development and plant immunity via repressing the SA signaling pathway [99]. Biotrophic pathogen evolves strategies to produce auxin or manipulate auxin signaling to suppress host SA signaling, thus contributing to its virulence [155]. Auxins limit the H_2_O_2_ levels through restraining H_2_O_2_ generation to be implicated in the regulation of stomatal opening. BRs are a unique class of plant steroid hormones that mediate growth and development. BRs signals are perceived and transduced by the receptor-like kinase BRASSINOSTEROID INSENSITIVE1 (BRI1) and BRI1-ASSOCIATED KINASE1 (BAK1). Pathogen *Pythium graminicola* manipulates plant BR pathway to antagonize SA- and GA-mediated defenses to promote its infection [156]. Meanwhile, BRs activate NADPH oxidase activity, which results in an accumulation of H_2_O_2_ levels in apoplast [157].

Phytohormones SA, ET, JA, ABA, and BRs play positive roles in regulating leaf senescence, whereas CK, auxins, and GA delay this process [11]. The contents of SA, JA, ET, and ABA increase during leaf senescence and promote this process [1], suggesting that these hormones could synergistically work on leaf senescence process, although many studies describe an antagonistic interaction between the SA and JA/ET/ABA pathways. The application of low concentrations of JA and SA leads to a synergistic effect on the JA- and SA-responsive genes *PDF1.2* and *PR1*, respectively. In addition, a high concentration of JA is required for the induction of H_2_O_2_ accumulation, thus JA might promote leaf senescence by partially interacting with ROS [99].

## 5. Roles of SA/ROS in Biotic and Abiotic Stresses

**Figure 3:**
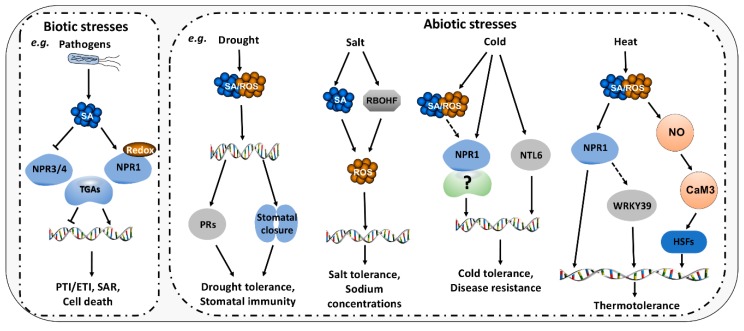
Roles of SA/ROS in Biotic and Abiotic Stresses. In biotic stresses, SA accumulation occurs after pathogen attack. Subsequently, SA binds to and activates NPR1 that interacts with TGAs to induce defense-related genes. Meanwhile, ROS is required for changes in redox status of NPR1. Besides, SA represses the transcriptional inhibition activities of NPR3/NPR4 to release defense-related genes. The production of SA/ROS is also triggered by various abiotic stresses including drought, salt cold and heat. SA/ROS further regulates NPR1-dependent or NPR1-independent gene expression. Plasma membrane-located NTL6 is relocated into the nuclear to induce a subset of PR genes in response to cold stress. Heat stress-induced ROS cause the accumulation of NO that activates CaM3 and results in DNA targeting of HSFs. TGA, TGACG motif-binding factor; PTI, PAMP-triggered immunity; ETI, Effector-triggered immunity; SAR, systemic acquired resistance; PRs, Pathogenesis-related genes; RBOHF, respiratory burst oxidase homolog; NTL6, NAC with transmembrane motif 1-like; NO, Nitric Oxide; CaM3, Calmodulin 3; HSFs, Heat shock transcription factors.

SA and ROS play important roles in the regulation of plant development as well as biotic and abiotic stress responses (Figure 3).

### 5.1. Roles of SA/ROS in Biotic Stresses

Biotic stresses (focused on pathogen attacks) cause changes of SA and ROS contents by multiple layers of transcriptional regulation (Figure 1). SA is perceived by its potential receptor NPR1 and NPR3/4 [20]. NPR1 or NPR3/4 interacts with a number of TGA TFs, including TGA2/5/6 to positively or negatively regulate SA-induced PR genes expression and disease resistance, respectively [20,158]. Pathogen-induced SA accumulation promotes the activity of NPR1 to further induce the expression of defense-related genes, while SA represses the transcriptional inhibition activities of NPR3/NPR4 to release defense-related genes. In plant-pathogen interactions, the production of ROS is one of the fastest physiological responses observed in plants after MAMP recognition by PRRs. ROS can induce Ca^2+^ influx into the cells, activate MAPK cascades, and regulate the redox of TFs, thus activate the expression of defense-related genes [159,160]. Rapid production of ROS leads to HR or HR-like necrosis that contributes to limiting the spread of biotrophs, but it generally benefits the necrotrophs infection [9,10].

### 5.2. Roles of SA/ROS in Abiotic Stresses

Drought, salt, cold, and heat are the most common adverse abiotic stresses that seriously influence plant growth and development. Water deficit increases the levels of SA/ROS, and drought stress induces SA-responsive PR genes [161], suggesting that SA/ROS might play a key role in drought tolerance. The mutation of a SIZ-type small ubiquitin-related modifier (SUMO) E3 ligase causes stomatal closure and then enhances drought tolerance by elevating SA/ROS accumulation [161]. It should be noted that plants can close stomata via SA/ROS signaling after PAMP recognition, termed as stomatal immunity [49,162], implying that SA/ROS are involved in drought stress and plant immunity.

SA is also involved in salt tolerance. The germination of *sid2* seeds is hypersensitive to salt stress. The endogenous H_2_O_2_ is elevated in wild type and *sid2* seeds under high salinity. However, the level of H_2_O_2_ is reduced after treatment with SA, suggesting that SA plays an essential role in seed germination under salt stress by balancing ROS [163]. The loss-of-function of RBOHF decreases salinity-induced ROS and increases sodium concentrations in root vasculature, indicating that RBOHF functions in salinity-induced vasculature-specific ROS accumulation and salt tolerance [164].

Cold stress also increases the levels of SA/ROS [165], indicative of a close link between cold stress and immune responses. In *Arabidopsis*, NTL6 has been demonstrated to be an important regulator in linking cold signals with pathogenesis [166]. Cold could enhance disease resistance by activating NTL6, which upregulates several PR genes by directly binding their promoters, such as *PR1*, *PR2*, and *PR5*. However, NTL6-mediated PR genes expression is independent of SA [166]. Recently, NPR1 was found to mediate a novel regulatory pathway in cold acclimation independently of SA and TGA [167]. However, the exogenous application of SA improves the cold tolerance in maize, cucumber, and rice [168].

Benzothiadiazole (BTH), an SA analogue, can induce thermosensitive genes, indicating that SA is involved in heat stress [169]. Application of BTH induces bacterial resistance in a NPR1-dependent manner [169]. *cpr5* (*constitutive expressor of PR genes*), a SA constitutive accumulation mutant, displays a high thermotolerance, while SA-deficient *NahG* transgenic plants exhibit an opposite phenotype. The overexpression of *WRKY39* enhances thermotolerance by upregulating SA-related genes [170]. ROS accumulation is required for heat stress responses in plants. Heat stress-induced ROS are mainly produced in chloroplasts by RBOHB and RBOHD. ROS causes the accumulation of nitric oxide (NO), which subsequently activates Calmodulin 3 (CaM3), and then enhances the binding activity of HSFs (heat shock transcription factors) [171].

## 6. Concluding Remarks

Numerous signaling components are involved in both leaf senescence and plant immunity, which suggests that these two events are not independent biological processes and share partial regulatory networks. It is apparent that SA and ROS play important and overlapping roles in leaf senescence and disease resistance. The co-players promote leaf senescence and immune response by converging on the SA and ROS pathways, which explains the phenomenon that the early-senescing mutants with increased SA and/or ROS levels are more resistant to biotrophic pathogens, but more susceptible to necrotrophic pathogens. The relationships between these two processes have not been systematically studied, despite outstanding progresses have been made over the past few decades in the individual field of leaf senescence and plant immunity. Based on our above-mentioned gene network analysis, it is reasonable to speculate that a large number of *SAG*s are also involved in regulating plant immunity, and vice versa. Thus, in-depth understandings of the cross-regulatory mechanisms between leaf senescence and plant immunity will facilitate the cultivation of high-yield and disease-resistant crops through molecular breeding strategy.

## Figures and Tables

**Table 1:** List of the genes that are involved in leaf senescence and plant immunity.

Family	Gene	AGI Code	SAG	Leaf Senescence	Resistance ^a^	Resistance ^b^
NAC	*ORE1*	AT5G39610	Y	Positive [28]	Positive [42]	
	*NTL9*	AT4G35580	Y	Positive [39]	Positive [43]	
	*NAC032*	AT1G77450	Y	Positive [40]	Positive [45]	
	*ATAF1*	AT1G01720	Y	Positive [41]	Positive [50,51]	Negative [44,52,53]
	*ANAC046/087*	AT3G04060/AT5G1827	Y	Positive [54]		
	*JUB1*	AT2G43000	Y	Negative [6]	Negative [55]	Positive [56]
	*NAC017/082/090*	AT1G34190/AT5G09330/AT5G22380	Y	Negative [57]		
	*NAC019/055/072*	AT1G52890/AT3G15500/AT4G27410	Y	Positive [58,59]	Negative [60]	Negative [61]
	*ORS1*	AT3G29035	Y	Positive [62]		
	*NTL4*	AT3G10500	Y	Positive [63,64]		
WRKY	*WRKY53*	AT4G23810	Y	Positive [29]	Positive [65]	
	*WRKY54/70*	AT2G40750/AT3G56400	Y	Negative [66]	Positive [67]	Negative [67]
	*WRKY6*	AT1G62300	Y	Positive [68]	Positive [68,69]	
	*WRKY22*	AT4G01250	N1	Positive [70]	Positive [71]	
	*WRKY18/40/60*	AT4G31800/AT1G80840/AT2G25000	Y	Negative [72]	Negative [73,74]	Positive [74]
	*WRKY75*	AT5G13080	Y	Positive [5]	Positive [5]	
bZIP	*GBF1*	AT4G36730	Y	Positive [75]	Positive [76]	
MYB	*LUX*	AT3G46640	Y	Negative [77]	Positive [78]	
TCP	*TCP19*	AT5G51910	N1	Negative [79]		
	*TCP20*	AT3G27010	Y	Negative [79]		
Non-TF	*SAG101*	AT5G14930	Y	Positive [80]	Positive [23]	
	*HLS1*	AT4G37580	N	Negative [81]		Positive [81]
	*JMJ16*	AT1G08620	Y	Negative [82]		
	*MPK3*	AT3G45640	N1	Positive [83]	Positive [84]	Negative [85]
	*MPK6*	AT2G43790	Y	Positive [83]	Positive [84]	Negative [85]
	*FTSH4*	AT2G26140	Y	Negative [86]		

^a^ Resistance ability to biotrophic and hemi-biotrophic pathogens. ^b^ Resistance ability to necrotrophic pathogens. N^1^ indicates that the gene is down-regulated during senescence. N represents that the transcriptional level of the gene exhibits no significant changes during senescence. The data of the reference [34] are used for SAGs identification.

**Table 2:** List of phytohormone signal components in leaf senescence and plant immunity.

Hormone	Component	Function	AGI Code	Role in Leaf Senescence	Role in Resistance ^a^	Role in Resistance ^b^
**ET**	EIN2	Involved in ethylene signal transduction	AT5G03280	Positive [100]	Ambiguous [42,101,102]	Negative [103]
	EIN3	Key transcription factor that initiates downstream transcriptional cascades for ethylene responses	AT3G20770	Positive [100]	Negative [102]	Positive [104]
	EIL1	EIN3-like 1	AT2G27050	Positive [100]	Negative [102]	Positive [104]
**JA**	COI1	Key component required for JA-induced transcriptional regulation	AT2G39940	Positive [105,106]	Negative [107]	Positive [21,108] Negative [103,109]
	JAZ7	Protein constitutes JA receptor complex with COI1	AT2G34600	Negative [110]	Positive [111]	Negative [111]
	MYC2	A MYC-related transcriptional activator interacted with JAZs	AT1G32640	Positive [112]	Negative [113]	Negative [114,115]
**SA**	ICS1	Required for SA biosynthesis	AT1G74710	Positive [5]	Positive [15]	Non [18]
	NPR1	Key regulator of SA-mediated disease resistance	AT1G64280	Positive [22]	Positive [20]	Non [21]
	EDS1	Component of R gene-mediated disease resistance	AT3G48090	Positive [22,25]	Positive [23]	Negative [24]
	PAD4	A lipase-like protein that is important for SA signaling.It can interact with EDS1	AT3G52430	Positive [22,25]	[23]	
**ABA**	PYL9	ABA receptor	AT1G01360	Positive [116]		
	SnRK2.8	A member of SNF1-related protein kinase involved in ABA signaling	AT1G78290	Positive [116]	Positive [117]	
	ABI5	A bZIP transcription factor involved in ABA signaling	AT2G36270	Positive [118]		Non [81]
**GA**	DELLAs	Repressor of GA responses and involved in GA-mediated signaling	AT1G14920	Negative [119]	Negative [120]	Positive [120]
**CK**	AHK3	Cytokinin receptor	AT1G27320	Positive [121]	Positive [122]	
	ARR2	A transcription factor involved in the cytokinin signaling pathway	AT4G16110	Non [123]	Positive [122]	
**Auxin**	TIR1	Auxin receptor	AT3G62980	Positive [124]		
	ARF2	An auxin response factor	AT5G62000	Positive [125,126]		
**BR**	BRI1	A plasma membrane localized leucine-rich repeat receptor kinase involved in brassinosteroid signal transduction	AT4G39400	Positive [11]		Negative [127]

^a^ Resistance ability to biotrophic and hemi-biotrophic pathogens. ^b^ Resistance ability to necrotrophic pathogens.
